# Functional Lumen Imaging Probe Measurement Post‐Pneumatic Dilation in Clinically Relevant Esophagogastric Junction Outlet Obstruction

**DOI:** 10.1111/nmo.70053

**Published:** 2025-04-24

**Authors:** James D. Miller, Zachary L. Mitchell, Abigail L. Ellington, Felicia A. Peoples, Steven B. Clayton

**Affiliations:** ^1^ Wake Forest University School of Medicine Winston‐Salem North Carolina USA; ^2^ Section on Gastroenterology Wake Forest University School of Medicine Winston‐Salem North Carolina USA

**Keywords:** Esophagogastric junction outlet obstruction, FLIP, pneumatic dilation, reduced esophageal opening

## Abstract

**Background:**

Pneumatic dilation (PD) is an effective treatment for disorders of reduced esophageal opening. Functional lumen impedance planimetry (FLIP) can effectively measure lower esophageal sphincter (LES) physiology compared to esophageal standards. The aim of this retrospective cohort analysis was to evaluate if FLIP measurements and esophageal opening classifications changed consistently with symptom improvement post‐PD. Also, the aim was to determine if post‐PD FLIP measurement correlated with the need for repeat dilation.

**Methods:**

Patients with clinically significant esophagogastric junction outlet obstruction (EGJOO) with reduced esophageal opening (REO) or borderline REO (BrEO) based on FLIP, timed barium esophagram (TBE), and manometry who underwent PD were included. Post‐PD FLIP measurements were taken immediately after PD during the same endoscopy encounter.

**Results:**

After PD, average distensibility index (DI) increased from 1.5 mm^2^/mmHg to 4.7 mm^2^/mmHg (*p* < 0.001) and diameter changed from 8.9 mm to 15.9 mm (*p* < 0.001). Average post‐dilation Eckardt score was 1.2, decreasing from an average pre‐dilation score of 6.25.

Of those requiring repeat dilations, average post‐dilation DI was 4.5 mm^2^/mmHg and diameter 16.4 mm, not statistically different from those that did not undergo repeat procedure (*p* = 0.79, 0.67, respectively). Post‐dilation esophageal openings were all NEO or BnEO. Average Eckardt score at 6–8 week follow‐up was not significantly different from those who did not require repeat dilation (1.4, *p* = 0.112).

**Conclusions:**

PD appears to be associated with improved esophageal opening and a significant change in both DI and diameter, consistent with an improved Eckardt score. Post‐dilation DI, diameter, esophageal opening pattern, and Eckardt score did not reveal a trend indicating the need for repeat dilation.

## Graphical abstract

In a retrospective cohort study of 29 EGJOO patients receiving PD, FLIP metrics and Eckardt score all improved after PD. In 6 patients requiring early repeat dilation for symptom recurrence, no difference in FLIP findings was identified.

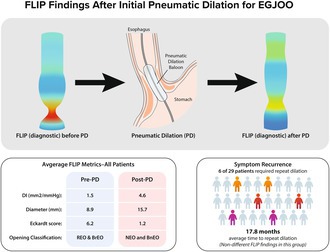

**Summary**

- Pneumatic dilation is effective for treatment of reduced esophageal opening.
- Improvementin FLIP measurements post‐dilation consistent with Eckardt score.
- FLIP did not reveal trend to predict need for repeat dilation.

## 1 Introduction

Pneumatic dilation (PD) has been shown to be an effective treatment for disorders of reduced esophageal opening, such as clinically relevant esophagogastric junction outlet obstruction (EGJOO) as well as achalasia [1, 2, 3]. Clinically relevant EGJOO is defined by high‐resolution manometry (HRIM) findings of elevated integrated relaxation pressure (IRP) of the lower esophageal sphincter (LES) with supportive evidence from either timed barium esophagram (TBE) and/or functional lumen imaging probe (FLIP), preserved peristalsis, and correlating symptoms of dysphagia [4, 5]. A previous study has shown symptom improvement from clinically relevant EGJOO post‐PD correlated with improved findings on TBE [6]. However, EGJOO has a variable disease course from spontaneous resolution to progression to achalasia, making management decisions largely symptom‐guided and individualized [7, 8, 9, 10, 11, 12, 13]. There remains limited data on EGJOO progression in the literature, specifically what findings may be predictive of treatment response and disease progression to achalasia.

FLIP has been recommended for use as adjunct testing to HRIM in the diagnosis of EGJOO [14]. Additional studies have shown utility in LES assessment perioperatively [15, 16]. Given that FLIP has been shown to be useful in predicting some procedure outcomes, certain measurements may guide outcome predictions after LES intervention, like PD [17, 18].

The aim of this study is to investigate the physiologic response to PD as measured by FLIP and determine the utility of these measurements in predicting the need for repeat dilation. We hypothesize that PD will be associated with a significant change in FLIP measurement and that post‐PD distensibility index (DI) will be lower in those needing repeat dilation.

## 2 Materials and Methods

### 2.1 Population

Patient data was obtained through retrospective chart review. Patients diagnosed with clinically relevant EGJOO by HRIM and confirmatory TBE and/or FLIP who underwent PD were included in the study. Further inclusion criteria for the study were that the patient (1) had documented LES measurements by FLIP within 1 year prior to PD without other definitive treatment in the interval and (2) underwent FLIP assessment immediately after PD in the same endoscopy encounter. Patients with additional anatomic features that could contribute to reduced esophageal emptying, i.e., stricture, ring, and web, were excluded from the study.

Patients were surveilled after PD with routine follow‐up at 6–8 weeks as available. Subsequent follow‐up with gastroenterologist was routine at 6–12 months or more frequently as indicated by symptom recurrence.

### 2.2 HRIM Protocol

Manometry was performed utilizing the ManoScan ESO high‐resolution manometry catheters system. ManoView software with high‐resolution esophageal color topography was used for data analysis. After confirming patients had performed at least 6 h of fasting before the test, the catheter was positioned to record from the hypopharynx to the stomach. The manometry was performed according to the Chicago Classification versions 3.0 and 4.0 protocol depending on when the study was performed. All relevant data was collected, including IRP, intrabolus pressure, distal contractile integral, LES basal sphincter pressure, distal latency, and presence of peristalsis. Abnormal studies were evaluated in agreement with the Chicago Classification version 4.0 criteria depending on when the study was performed. EGJOO, as defined by the Chicago criteria v.4, has an elevated IRP obtained by HRIM with a cutoff of > 15 mmHg.

### 2.3 TBE Protocol

Patients were administered 240 mL (8 0z) of low‐density barium in the standing position; two‐on‐one spot films were obtained at 1 and 5 min to assess liquid emptying. Next, the esophagus was cleared with water followed by ingestion of a 13‐mm barium tablet. Tablet passage was evaluated after 5 min, with abnormal testing being tablet retention. Patients with a barium column height of > 5 cm after 1 min in the distal esophagus, > 2 cm after 5 min, and/or pill retention after 5 min were included in the study. The following parameters were recorded: barium height at 1 and 5 min in centimeters, barium width at 1 and 5 min in centimeters, and tablet retention.

### 2.4 FLIP Protocol

The FLIP studies were performed by inserting the 16 cm catheter (EndoFLIP EF‐322 N; Medtronic Inc., Shoreview, MN) orally during endoscopy. Once the catheter was inserted, positioning was determined by identification of the waist of the catheter formed by the LES on real‐time 3D modeling once the catheter had been inflated to a low volume, typically 20–30 mL. Positioning of the catheter throughout the study is maintained by visual identification of the LES “waist” of the catheter.

The FLIP records 16 cross‐sectional areas along the length of the catheter and one intra‐balloon pressure at a 10‐Hz sampling rate during distension. The driver of the catheter controls the distension by altering the volume contained within the balloon. From cross‐sectional area measurements, the diameter of the balloon is calculated. The cross‐sectional area and the pressure within the balloon vary with volume within the balloon as well as contraction cycles and respiratory activity, so stable volume filling within the balloon must be maintained to ensure accurate measurements over time. The volume was increased by 10 mL increments in a stepwise manner up to 70 mL with significant duration between steps to ensure accurate measurements of pressure for each volume per suggested manufacturer. At our institution, our initial protocol stopped the balloon fill at 60 mL in accordance with the Medtronic white paper but later was modified to continue filling to 70 mL fill volume unless the balloon pressure exceeded 80 mmHg per manufacturer recommendations.

Reduced esophageal opening (REO) was defined as DI of < 2.0 mm^2^/mmHg and a maximum diameter of < 12 mm. Borderline reduced esophageal opening (BrEO) was defined as DI < 2.0 mm^2^/mmHg or maximum diameter < 14 mm. Borderline Normal Reduced Esophageal Opening (BnEO) was defined as a maximum diameter 14–16 mm or DI < 2.0 mm^2^/mmHg and diameter ≥ 16 mm. Normal Esophageal Opening (NEO) was defined as DI > 2.0 mm^2^/mmHg and diameter ≥ 16 mm [19].

### 2.5 PD Protocol

Pneumatic dilatation was performed using the Rigiflex balloon system (Boston Scientific, Marlborough, MA, USA). These noncompliant polyethylene balloons are available in three diameters (30, 35, and 40 mm), mounted on a flexible catheter placed over a guidewire. Endoscopy is performed, and the LES is identified and sometimes marked with a radiopaque marker fluoroscopically, depending on the endoscopist's preference. Pneumatic dilatation was performed under fluoroscopic guidance. The balloon is positioned across the LES and gradually inflated until the “waist” is effaced/obliterated. The pressure required is usually 7–15 p.s.i. of air, which is held for 1 min while monitoring the balloon position fluoroscopically. All patients included were undergoing initial PD, and only the 30 mm balloon was used. If repeat PD was performed due to symptom recurrence, the next larger balloon size available was used in a graded dilation protocol, all 35 mm in these cases.

### 2.6 Statistical Analysis

Quantitative data were expressed as mean and standard deviation. Analysis of quantitative parametric data was assessed with student's *t* test. For categorical data, a chi‐squared test was used. For the purposes of this study, a *p* value of < 0.05 was considered statistically significant.

## 3 Results

A total of 29 patients with clinically relevant EGJOO who underwent PD had qualifying FLIP testing and were included in the study. Their demographic characteristics are summarized in Table 1.

**TABLE 1:** Patient characteristics and EGJ opening classifications.

	Average	(±)
Age	65.1	14.1
% Female	48	
BMI (kg/m^2^)	28.7	5.6

Opening classification	*N*	
Pre‐dilation		
REO	22	
BrEO	7	
Post‐dilation		
REO	2	
BrEO	3	
BnEO	8	
NEO	16	

All patients with clinically relevant EGJOO had dysphagia as their main complaint. The majority (*n* = 22) met criteria for REO and 7 met criteria for BrEO. Following PD, 27 patients (97%) had improvement in their opening classification while 2 did not, continuing to meet criteria for REO. These 2 patients, both dilated with a 30 mm balloon, demonstrated residual abnormal findings on post‐dilation TBE but showed post‐PD improvement in Eckardt score from 9 to 1 and 9 to 0, respectively.

**FIGURE 1:**
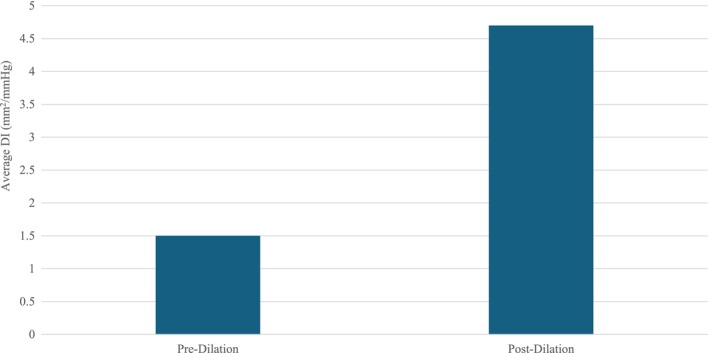
Average distensibility index in all patients pre and post‐pneumatic dilation.

**FIGURE 2:**
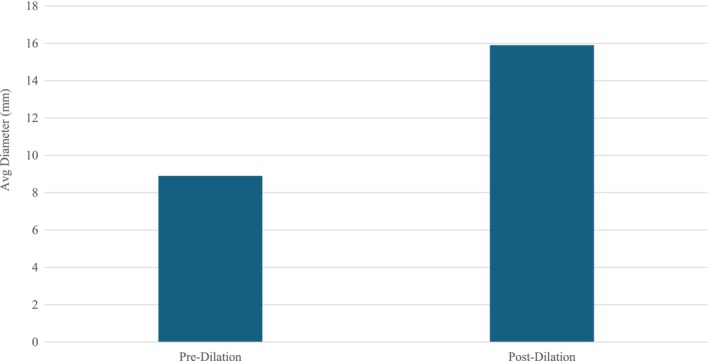
Average diameter in patients pre‐and post‐pneumatic dilation.

Average LES DI of all patients pre‐PD was 1.5 mm^2^/mmHg. Post‐PD, average DI was 4.6 mm^2^/mmHg (*p* < 0.001) (Figure 1). Average LES Diameter of all patients pre‐PD was 8.9 mm. Post‐PD, average Diameter was 15.7 mm (*p* < 0.001) (Figure 2).

15 patients had Eckardt Score documented at their 6–8 week follow‐up appointment. Average pre‐dilation Eckardt score was 6.25. Average post‐dilation Eckardt score was 1.2 (*p* < 0.001).

**FIGURE 3:**
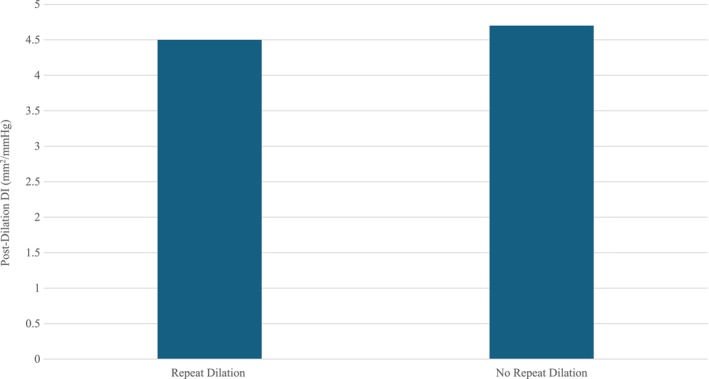
Initial average post‐pneumatic dilation distensibility index grouped by need for repeat dilation.

**FIGURE 4:**
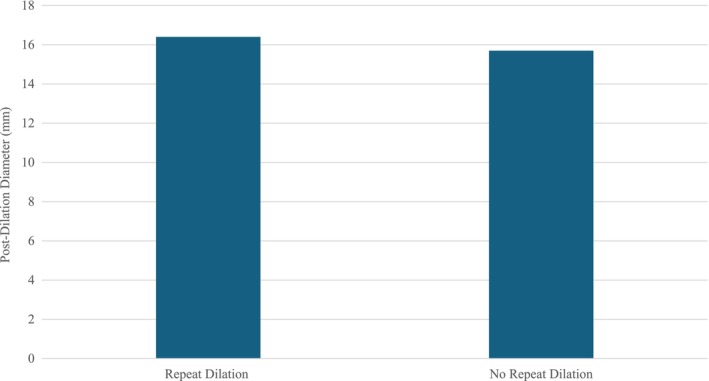
Initial average post‐pneumatic dilation diameter grouped by a need for repeat dilation.

Six patients experienced symptom recurrence requiring repeat dilation with an average interval of 17.8 months between initial PD (median 17.5 months). Sixty‐seven percent underwent repeat PD, 33% Savory dilation. Of those that required repeat dilations, the average post‐dilation DI was 4.3 mm^2^/mmHg, and diameter 15.5 mm. Of those that did not require repeat dilation, the average post‐dilation DI was 4.7 mm^2^/mmHg and the average diameter was 15.7 mm (*p* = 0.64, 0.96) (Figures 3 and 4). Post‐dilation esophageal opening classifications were all NEO or BnEO. Pre‐dilation opening criteria all changed post‐dilation. The average post‐dilation Eckardt score at 6–8 week follow‐up for patients requiring repeat dilation was 1.4. The average post‐dilation Eckardt score for patients not requiring repeat dilation was 0.7 (*p* = 0.112). The median follow‐up duration of all patients was 23 months.

## 4 Discussion

Our study aimed to evaluate FLIP measurements surrounding PD, which showed significant changes in both DI and Diameter immediately following PD (*p* < 0.001, < 0.001). We also identified improvement in Eckardt score post‐PD as previously shown (*p* < 0.001). We did not observe a significant difference in pre‐ or post‐PD DI, diameter, opening classification, or Eckardt score among patients needing repeat dilation to suggest a long‐term predictive trend (*p* = 0.79, 0.67, 0.112).

The clinical role for FLIP has thus far been limited in practice guidelines to use as an adjunct test in the diagnosis of EGJOO after completion of first‐line testing with HRIM [14]. However, additional literature has supported the use of FLIP to assess LES distensibility post‐operatively in fundoplication and esophagectomy [16, 20, 21]. With FLIP measurement change identified in these settings, it is not surprising that DI, diameter, and opening classification changed significantly immediately post‐PD. Similarly, symptoms of dysphagia from EGJOO related to LES pathology and no impaired peristalsis would also be expected to improve after intervention, as shown previously [6]. We observed a significant change in Eckardt score post‐PD along with coinciding FLIP changes.

Ideal treatment is unknown for clinically relevant EGJOO and is being studied. There are many treatment options available to treat the functional obstruction at the esophagogastric opening from a poorly relaxing LES in both patients with EGJOO and achalasia, and many patients may require repeat procedures to achieve clinical improvement [7, 12, 13]. Initial symptomatic improvements in achalasia patients was shown by Vaezi et al. to be a poor predictor of procedural success when compared to objective measures, such as TBE [22]. Similarly, we observed improved symptoms and significantly decreased Eckardt scores at 6–8 week follow‐up even in those patients with early symptom recurrence. Therefore, a test able to predict the long‐term effectiveness of procedural treatments for reduced esophageal opening would be highly beneficial in long‐term management of these patients.

Previous studies have investigated the utility of FLIP in the prediction of procedural treatment outcomes with some promising but inconsistent findings [17, 18, 20, 23, 24]. For example, the degree of change in DI post‐PD has been shown by Wu et al. to correlate with immediate post‐procedural symptom improvement in achalasia patients [25]. Similarly, we identified a significant change in average DI and improvement in Eckardt score at short‐term follow‐up. However, our study identified that even patients requiring short interval repeat dilation showed the same improvements without a clear trend to predict this from FLIP findings. Additionally, our study focused on EGJOO as opposed to achalasia, included the determination of esophageal opening classification for consistency with up‐to‐date nomenclature, and used a 70 mL FLIP balloon maximum inflation volume for more complete distention compared to 40 mL in the above study.

We observed consistent change in esophageal opening classification post‐PD that provides further validation to the clinical relevance of these subclassifications of EGJOO. All symptomatic patients initially having either REO or BrEO classification on FLIP support the clinical distinction between “normal” and “reduced” opening. Recent studies utilized normal post‐procedural esophageal opening as an indicator of successful treatment and found it to be associated with symptom improvement in achalasia [26, 27]. However, we did not find a significant difference in post‐PD opening classifications among those needing repeat dilation, but our sample size of 29 patients should be noted.

Future investigation to improve upon the limitations of this study may include extended longitudinal follow‐up and a consistent protocol to obtain repeat FLIP with symptom recurrence prior to repeat PD. This would allow identification of the degree of DI, diameter, and opening classification change that occurs over time. A prior study had found no development of achalasia following PD for EGJOO with a mean follow‐up of 1.7 years [6]. Given the need for repeat dilation at an average interval of ~1.5 years in our cohort, follow‐up FLIP measurement at the time of symptom return may provide further evidence of the mechanism of progression to achalasia.

Our study had limitations related to its nature as a retrospective chart review. All diagnoses of EGJOO were made by a single clinician interpreting HRIM, FLIP, and TBE findings. Our sample size was limited to 29 and could be subject to statistical variance due to the small sample size; however, it was similar in size to related studies previously published. The limited number of patients who presented for follow‐up for gathering Eckardt score, and further reduced in the repeat dilation group, may also limit the ability to detect the significant change by type II error. Further, the decision to undergo repeat dilation was made by the clinician based on symptom recurrence. Additional FLIP measurements at the time of follow‐up in patients who met the indication for repeat endoscopy would be beneficial to evaluating changes in esophageal physiologic parameters that could predict symptom recurrence. The timeline of the retrospective study also limited the opportunity for additional patients to have symptom recurrence, as the most recent PD occurred only 2 years prior to study initiation. Given that symptom recurrence may happen as far as 5 years post‐PD in some studies, limiting the inclusion criteria to more distant PD may increase the repeat dilation group size of future studies.

In conclusion, our study demonstrated that FLIP measurements change significantly post‐PD with symptom improvement and change in esophageal opening classification. We did not observe a significant difference in post‐PD FLIP measurements or opening classification in patients needing repeat dilation; however, a larger study is needed to determine the predictive utility of such metrics.

## Author Contributions

J.D.M. designed the study, performed data collection, major analysis, and drafted/revised the manuscript. Z.L.M. performed data analysis. A.L.E. and F.A.P. contributed to manuscript edits. S.B.C. performed conception and design, analysis and interpretation of the data, drafting of the manuscript, and final approval of the article.

## Conflicts of Interest

The authors declare no conflicts of interest.

## Data Availability

The data that support the findings of this study are available from the corresponding author upon reasonable request.
